# Phosphodiesterase-5 inhibition preserves renal hemodynamics and function in mice with diabetic kidney disease by modulating miR-22 and BMP7

**DOI:** 10.1038/srep44584

**Published:** 2017-03-15

**Authors:** Riccardo Pofi, Daniela Fiore, Rita De Gaetano, Giuseppe Panio, Daniele Gianfrilli, Carlotta Pozza, Federica Barbagallo, Yang Kevin Xiang, Konstantinos Giannakakis, Susanna Morano, Andrea Lenzi, Fabio Naro, Andrea M. Isidori, Mary Anna Venneri

**Affiliations:** 1 Department of Experimental Medicine, Sapienza University of Rome, Italy; 2 Department of Anatomical, Histological, Forensic and Orthopedic Sciences, Sapienza University of Rome, Italy; 3 Department of Pharmacology, University of California at Davis, California, USA; 4 Department of Radiology, Oncology, Radiology and Pathology, Sapienza University of Rome, Italy

## Abstract

Diabetic Nephropathy (DN) is the leading cause of end-stage renal disease. Preclinical and experimental studies show that PDE5 inhibitors (PDE5is) exert protective effects in DN improving perivascular inflammation. Using a mouse model of diabetic kidney injury we investigated the protective proprieties of PDE5is on renal hemodynamics and the molecular mechanisms involved. PDE5i treatment prevented the development of DN-related hypertension (*P* < 0.001), the increase of urine albumin creatinine ratio (*P* < 0.01), the fall in glomerular filtration rate (*P* < 0.001), and improved renal resistive index (*P* < 0.001) and kidney microcirculation. Moreover PDE5i attenuated the rise of nephropathy biomarkers, soluble urokinase-type plasminogen activator receptor, suPAR and neutrophil gelatinase-associated lipocalin, NGAL. In treated animals, blood vessel perfusion was improved and vascular leakage reduced, suggesting preserved renal endothelium integrity, as confirmed by higher capillary density, number of CD31^+^ cells and pericyte coverage. Analysis of the mechanisms involved revealed the induction of bone morphogenetic protein-7 (BMP7) expression, a critical regulator of angiogenesis and kidney homeostasis, through a PDE5i-dependent downregulation of miR-22. In conclusion PDE5i slows the progression of DN in mice, improving hemodynamic parameters and vessel integrity. Regulation of miR-22/BMP7, an unknown mechanism of PDE5is in nephrovascular protection, might represent a novel therapeutic option for treatment of diabetic complications.

The vascular complications of diabetes dramatically increase the disease’s morbidity and mortality of patients. One third of diabetes patients suffers from diabetic nephropathy (DN)^1^, a leading cause of end-stage renal disease^2^,^3^. To date, effective treatment in halting or reversing the natural progression of DN remains uncertain^4^,^5^,^6^,^7^,^8^.

As with most diabetes-associated co-morbidities, the pathophysiology is multifactorial and the molecular pathways involved are complex. Glomerular endothelial cell (EC) injury plays a major role in the development and progression of diabetic kidney disease, that is considered the result of converging hemodynamic and metabolic insults^9^. In kidney, phosphodiesterase type 5 (PDE5), the cyclic guanosine monophosphate (cGMP) hydrolyzing enzyme, is expressed in the glomeruli, mesangial cells, cortical tubules, inner medullary collecting duct and plays a critical role in the regulation of excretory function^10^. In diabetes, glomerular cGMP production is decreased, PDE5 activity is increased^11^ and changes in the cGMP-NO pathway leads to a rise in intra-glomerular pressure^12^. PDE5 inhibitors (PDE5is) may have an active role in the management of DN^13^ by reducing glomerulosclerosis and proteinuria and improving vascular inflammation and podocyte count in experimental diabetes models^14^,^15^,^16^,^17^. These findings, together with the modulation of inflammatory^14^,^18^ and angiogenic mediators, and endothelial function^19^,^20^ in murine and human models, support the use of PDE5i to prevent complications of the diabetic kidney.

Intriguingly, sildenafil, a potent PDE5i, was also associated with improved kidney function in patients with pulmonary arterial hypertension^21^ and in type-2 diabetes (T2DM) patients^22^. However, little information is available on the effect of PDE5is on the progression of nephrovascular impairment in DN. Furthermore, the molecular factors involved in nephroprotection have not yet been characterized.

The renal resistive index (RRI), measured in the interlobar arteries of both kidneys by renal color Doppler ultrasound (RDU), offers a measure of intraparenchymal renal resistances, which have been correlated with DN progression in humans^23^,^24^,^25^.

The aim of this study is to identify the targets of PDE5is in DN and the mechanisms involved in renal protection, monitoring *in vivo* kidney flow parameters by RDU and analyzing histological, perfusion, cellular and molecular modulation.

## Materials and Methods

### Animal model

12-week-old male CD1 mice were randomly assigned to one of four groups (8 mice in each group): vehicle (CTRL), sildenafil (SILD), streptozotocin (STZ) or streptozotocin + sildenafil (STZ + SILD). Diabetes was induced using a single intraperitoneal injection (IP) of streptozotocin (STZ: 150 mg/kg, Sigma Aldrich). We used high-dose STZ protocol to induce insulin-dependent diabetes mellitus in mice because of its toxic effects on islet beta cells^18^. To reduce STZ acute renal toxicity we pre-treated mice with the SGLT-2 inhibitor phlorizin, as previously shown^26^. After 3 days of STZ-diabetic induction, sildenafil citrate (1.6 mg/kg, SILD: Viagra, Pfizer in saline 0.3% aqueous solution of DMSO) was administered IP daily, for 4 weeks. Appropriate vehicle controls were performed for each treatment (STZ and/or sildenafil). All STZ-induced animals were supplied with 10% sucrose water for 72 h after STZ to counteract post-injection hypoglycemia. Body weight and food consumption were monitored every 2 weeks in all animals (data not shown). For the time course flow cytometry study, 12-week-old male CD1 mice were randomly assigned (4 mice to each of CTRL, SILD, STZ, STZ + SILD) and analyzed at 3 time points. All mice were kept in a pathogen-free facility. All experiments were performed in accordance with Italian law (Law Decree 2010/63EU) and the study was approved by the Sapienza University’s Animal Research Ethics Committee and by the Italian Ministry of Health (165/2016-PR). Housing of one mouse per cage allowed individual measurement of food and water intake and urine output. A drop of tail blood after 3 h fasting was used to monitor glucose concentrations by MediSense Precision Plus kit (Abbott Diagnostics, Melbourne, Victoria, Australia). Criteria for euthanasia were based on an independent assessment by a veterinarian according to AAALAC guidelines.

### Clinical and laboratory investigation

The tail artery cuff method was used to measure MAP and heart rate (HR). Baseline and after sildenafil treatment blood glucose, triglycerides, creatinine, glycosuria, albuminuria, creatininuria were assessed by Siemens Advia 1800 analyzer. SuPAR/CD87 levels were measured by ELISA kit (Cohesion Biosciences).

### Renal artery Color Doppler Ultrasound

Renal Doppler ultrasound was performed using a Philips IU22™ ultrasound system equipped with a Broadband Linear Array Transducer 17 MHz extended frequency range. Renal Doppler blood flow was obtained taking the mean value of 3 different evaluations: peripheral arteries, mesorenal arteries and renal artery origin over both kidneys, guided by color flow mapping. These evaluations were performed at baseline and during treatment. A single investigator blinded to the treatment performed the Doppler ultrasound examination. Doppler flow was measured using an anterior approach. The measured parameters were: kidney length and volume, peak-systolic velocity (PSV), end-diastolic velocity (EDV), mean diastolic velocity (MDV) renal resistive index (RRI), acceleration time (AT) and HR. RRI was calculated as (PSV-EDV)/PSV. PSV and EDV are expressed as cm/s. Kidney volume was calculated with the Ellipsoid formula.

### *In vivo* endothelial permeability measurements

EC permeability in renal tissue was estimated using vascular tracers of different molecular weights, FITC dextrans and Evan’s Blue (Sigma Aldrich). Mice were injected through the tail vein with 0.2 ml of 0.9% saline solution containing 200 mg/ml of 40 kDa dextran or 1% of Evan’s Blue (0.96 kDa). The animals were euthanatized thirty (dextran) or sixty (Evan’s blue) minutes after injection of the dyes. Evan’s Blue extravasation was quantified by incubating 100 mg of renal tissue for 24 h at 55 °C with 500 μL formamide to extract the extravasated dye; optical density was then measured at 610 nm and converted into ng of dye per mg of tissue. The experiment was repeated in triplicate.

Tissues from FITC dextrans-injected mice were processed for immunofluorescence analysis. Kidneys were obtained from surgical resections, embedded in OCT (Tissue-Tek, Torrance, CA) compound and snap-frozen. Sections were cut into 20-μm thickness by cryostat (Leica Microsystems, Germany). Extravasation of FITC–dextran produced a fluorescence outside the vessels that was classified as weak (+), medium (++), strong (+++) or diffuse (++++) according to intensity and profile. Weak extravasation was defined as a faint cloud associated with one vessel leakage site; medium extravasation was defined as a bright cloud clearly associated with one vessel leakage site; strong extravasation was defined as a bright cloud associated with more than one vessel; diffuse extravasation was defined as an extensive leakage of the tracer in the interstitial perivascular space without any clear association with disrupted vessels.

### Flow cytometry

The whole kidneys were reduced to single-cell suspensions by two digestions with collagenase type 2 (Sigma) and collagenase/dispase (Roche). The preparations were filtered through a 40 μm cell strainer (Becton Dickenson) and washed. The resulting single cells were collected, blocked in 5% v/v serum in PBS, centrifuged and then stained with the following antibodies: anti-CD31 (BD) and anti-CD45 (BD). Cells were labeled using 7-amino-actinomyocin D before analysis to detect necrotic cells. All samples were collected by a CyAn™ ADP cytometer (DAKO). A biexponential analysis was performed using Summit V4.3 software and FlowJo X (Treestar) software.

### Histological and immunofluorescence studies

Tissues were processed for cryosectioning or paraffin-embedding using standard methods. Kidney slices were stained with hematoxylin/eosin and PAS. Images were acquired using a bright field Aperto CS scanner and stored for subsequent analysis. Image Scope^®^ software was used to measure glomerular size, cellularity and mesangial matrix expansion and deposition. A single-blind operator performed all analyses. Glomerular diameters were measured in all visible glomeruli in the histological section by taking the maximum diameter from the hilum to the end of the Bowman’s capsule. Glomerular cellularity and mesangial matrix deposition were assessed through a specific program quantified by Image Scope algorithm (Positive pixel count version 8.100). Frozen slices were pre-blocked with serum and incubated with the following antibodies: rat anti-CD31 (eBioscience) followed by donkey anti–rat FITC–conjugated antibodies (Jackson Immunology), rabbit anti-NG2 followed by donkey anti–rabbit Cy5–conjugated antibodies. Images were analyzed with a Leica confocal microscope (TCS-SP2) and by Image-J software (NIH, Bethesda, USA). Capillary density was quantified from at least five high-power fields (×40) per slide, two slides per animal (3 animals per group). Sections were randomly selected and analyzed under blinded conditions.

### MicroRNA quantification

Kidney samples were homogenized with Trizol. Total RNA was purified and miRNA was extracted using a miRNA Isolation Kit (Qiagen). 10 ng of RNA were reverse transcribed using TaqMan MicroRNA Reverse Transcription Kit (Applied Biosystems). For cDNA synthesis, the reaction mixture was incubated at 16 °C for 30 minutes and then at 42 °C for 30 minutes; the enzyme was then inactivated at 85 °C for 5 min. 1.5 μL of the cDNA solution was then amplified using TaqMan Universal PCR Master Mix (Applied Biosystems), with miRNA-specific primer/probe (hsa-miR-22 000398, Thermo Fischer). The quantitative PCR was run on an ABI PRISM 7500 Real-time PCR system (Applied Biosystems) with an initial denaturation step at 95 °C for 10 minutes, followed by 40 cycles with a denaturation step at 95 °C for 15 seconds and an annealing/elongation step at 60 °C for 60 seconds. Each sample was run in triplicate for analysis. The miRNA expression levels were normalized to U6 snRNA (001973, Thermo Fischer).

### NGAL, TGFβ, BMP7 quantification

RNA was extracted using the Trizol protocol followed by DNase digestion to remove any contaminating genomic DNA. Total RNA concentration and purity were estimated using the Nanodrop ND-1000 spectrophotometer (Thermo Fisher SCIENTIFIC). 1 μg of RNA from each sample was reverse transcribed using the high capacity cDNA reverse transcription kit (Applied Biosystem). cDNA served as the template for subsequent PCR amplification with the following primers: mNGAL Fw 5-CTCAGAACTTGATCCCTGCC-3 and mNGAL Rw 5-TCCTTGAGGCCCAGAGACTT-3; mTGFβ1 Fw: TGACGTCACTGGAGTTGTACGG and mTGFβ1 Rw: GGTTCATGTCATGGATGGTGC; mBMP7 Fw 5-TGAGCTTCGTCAACCTAGTG-3 and mBMP7 Rw 5-TCCTTATAGATCCTGAATTCGG-3; 18S Fw 5-CCAGTAAGTGCGGGTCATAAGC-3 and Rw 5-AACGATCCAATCGGTAGTAGCG-3; mGAPDH Fw 5-AGGTCGGAGTCAACGGATTT-3 and mGAPDH Rw 5-GTGATGGCATGGACTGTGGT-3 as the internal control. Quantitative PCR (GoTaq, qPCR master mix, Promega) was run on an ABI PRISM 7500 Real-time PCR system (Applied Biosystems) with an initial denaturation step at 95 °C for 10 minutes, followed by 40 cycles with a denaturation step at 95 °C for 15 seconds and an annealing/elongation step at 60 °C for 60 seconds. Each sample was run in triplicate for analysis.

### Cell culture and treatment

Primary cultures of human umbilical vein endothelial cells (HUVECs) were obtained from ATCC and cultured as previously described^27^. For PDE5 hyperexpression, 10^5^ TU/ml of pCCL.sin.cPPT.hPGK.mPDE5 lentiviral vector (LV) was used to transduce HUVECs. To generate pCCL.sin.cPPT.hPGK.mPDE5, PDE5A was amplified from whole murine embryonic RNA by RT-PCR using a reverse primer and cloned between AgeI (5′) and SalI (3′) restriction sites into pCCL.sin.cPPT.hPGK. VSV-pseudotyped LV pCCL.sin.cPPT.hPGK.mPDE5 concentrated stocks were produced and titrated as described^27^. Transduced stable cells (PGK-PDE5) were used 10 days after infection. Cells were maintained for 24 hours in the presence of sildenafil (5 μM) or vehicle and analyzed for miR-22 expression.

### Statistical Analysis

All statistical analyses were carried out in accordance with a predetermined statistical analysis plan. The change from baseline was the post-baseline value minus the baseline value. The normality of distribution for all measurements at all time points was assessed by Shapiro-Wilk’s test (p > 0.05). The changes from baseline to week 4 were also analyzed with an ANOVA model that included the baseline as the covariate and treatment as the fixed effect. The ANOVA model used the last observation carried forward principle. Data are reported as mean with 95% Confidence Interval (95% CI) unless otherwise specified, whereas variables are reported as mean ± SD. A two-sided *P*-value of less than 0.05 was regarded as significant. Pearson’s correlation test was used to measure any linear association between variables. All analyses were performed using SPSS 18.0 (SPSS, Inc).

## Results

### Effect of sildenafil on metabolism and renal function of diabetic mice

**Figure 1:**
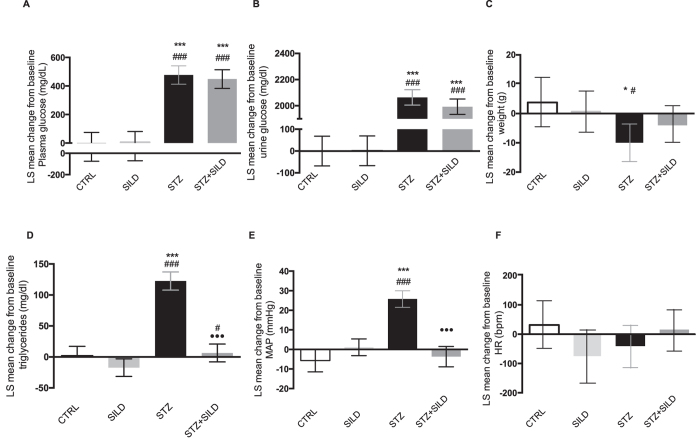
LS mean change from the baseline in: (**A**) plasma glucose, (**B**) urine glucose, (**C**) weight, (**D**) triglycerides, (**E**) MAP, (**F**) HR in CTRL, SILD, STZ and STZ + SILD mice. Error bars represent upper and lower 95% CI; *P < 0.05, ***P < 0.001 vs. CTRL; ^#^*P* < 0.05, ^###^*P* < 0.001 vs. SILD; ^•••^*P* < 0.001 vs. STZ.

All mice receiving streptozotocin (STZ and STZ + SILD groups) exhibited chronic hyperglycemia and glycosuria. The mean change in blood glucose levels from baseline in STZ and STZ + SILD mice was respectively 476.93 mg/dl (95% CI: 412.43 to 541.42) and 448.65 mg/dl (95% CI: 382.92 to 514.37), (Fig. 1A). The mean change in urine glucose levels was 2063.76 mg/dl (95% CI: 2005.07 to 2122.44) and 1992.45 mg/dl (95% CI: 1933.72 to 2051.18), (Fig. 1B). Blood and urine glucose levels were similar in sildenafil- and vehicle-treated diabetic mice. No changes were found in the non-diabetic groups (CTRL and SILD).

Induction of diabetes with STZ produced a weight loss that was attenuated by sildenafil. The mean change from baseline was −10.05 g (95% CI: −16.51 to −3.60) in STZ and −3.80 g (95% CI: −9.87 to 2.26) in STZ + SILD. Least square (LS) mean differences among groups were as follows: STZ vs. CTRL −13.91 (95% CI: −25.54 to −2.29), *P* = 0.024; STZ vs. SILD −10.61 (95% CI: −19.97 to −1.26), *P* = 0.030. Interestingly, sildenafil reduced weight loss to a level not statistically different from the non-diabetic groups: STZ + SILD vs. CTRL −7.66 g (95% CI: −18.40 to 3.07), *P* = 0.141, STZ + SILD vs. SILD −4.36 g (95% CI: −13.55 to 4.82), *P* = 0.311, (Fig. 1C).

Hypertriglyceridemia in diabetic mice improved after sildenafil, with a LS mean difference between STZ + SILD and STZ of −116.16 mg/dl (95% CI: −136.05 to −96.97), *P* < 0.001 (Fig. 1D). MAP was higher in diabetic mice and lower in sildenafil-treated animals. The mean change from baseline was 25.75 mmHg (95% CI: 21.53 to 29.96) and −3.68 mmHg (95% CI: −8.89 to 1.52) respectively in STZ and STZ + SILD; LS mean difference −29.42 mmHg (95% CI: −36.01 to −22.83, *P* < 0.001). In contrast, there were no differences in blood pressure between STZ + SILD and non-diabetic mice (Fig. 1E).

**Figure 2:**
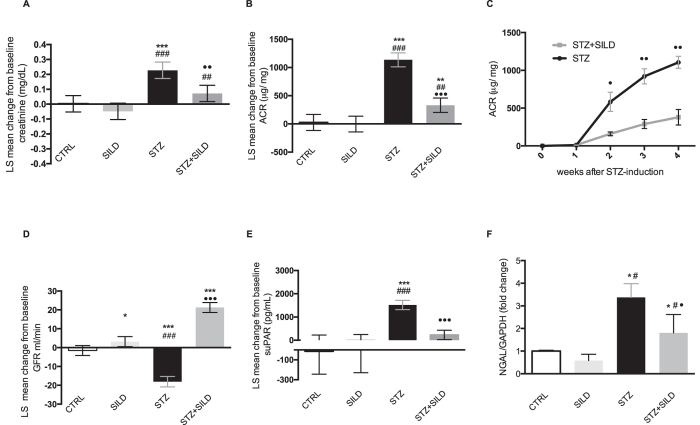
LS mean change from the baseline in: (**A**) creatinine, (**B**) ACR in CTRL, SILD, STZ and STZ + SILD mice. Error bars represent upper and lower 95% CI; **P < 0.01, ***P < 0.001 vs. CTRL; ^##^*P* < 0.01, ^###^*P* < 0.001 vs. SILD; ^••^*P* < 0.01, ^•••^*P* < 0.001 vs. STZ. (**C**) quantification (mean ± SE) of ACR over time course, in STZ and STZ + SILD groups, ^••^*P* < 0.05, ^••^*P* < 0.01 between groups. LS mean change from the baseline of (**D**) GFR, and (**E**) suPAR, in CTRL, SILD, STZ and STZ + SILD mice. Error bars represent upper and lower 95% CI; *P < 0.05, ***P < 0.001 vs. CTRL; ^###^*P* < 0.001 vs. SILD; ^•••^*P* < 0.001 vs. STZ. (**F**) relative expression of NGAL, mean ± SD, in CTRL, SILD, STZ and STZ + SILD mice. *P < 0.05 vs. CTRL; ^#^*P* < 0.05 vs. SILD; ^•^*P* < 0.05 vs. STZ.

There was no difference in HR between the four groups (Fig. 1F). In diabetic mice, elevated serum creatinine was lowered by sildenafil treatment: mean change from baseline 0.23 mg/dl (95% CI: 0.17 to 0.28) in STZ and 0.07 mg/dl (95% CI: 0.02 to 0.13) in STZ + SILD; LS mean difference −0.15 mg/dl (95% CI: 0.23 to −0.08), *P* < 0.01 (Fig. 2A).

As expected, the urinary albumin/creatinine ratio (ACR) increased in diabetic mice during the observation period: mean change from baseline 1135.58 μg/mg (95% CI: 1012.00 to 1259.15) in STZ and 331.65 μg/mg (95% CI: 204.80 to 458.49) in STZ + SILD. Sildenafil treatment significantly attenuated ACR: LS mean difference −803.93 μg/mg (95% CI: −985.84 to −622.01), *P* < 0.001 (Fig. 2B,C). Even when corrected for MAP, the LS mean difference was −893.48 μg/mg (95% CI: −1343.12 to −443.84), *P* = 0.002.

GFR was estimated using a single bolus injection of FITC-inulin. The mean change from baseline was unremarkable in the CTRL group, but significantly different in STZ: −18.07 ml/min (95% CI: −20.84 to −15.30), and in STZ + SILD: 21.28 ml/min (95% CI: 18.63 to 23.93). Sildenafil protected against the decline in renal function induced by diabetes: LS mean difference was 39.35 mL/min (95% CI: 35.43 to 43.28), *P* < 0.001, (Fig. 2D). This effect was retained even when adjusted for MAP: LS mean difference was 39.22 mL/min (95% CI: 19.18 to 59.35), *P* = 0.002.

Soluble urokinase-type plasminogen activator receptor (suPAR), a candidate biomarker for incipient chronic kidney disease, was markedly elevated in STZ mice: mean change from baseline 1516.58 pg/ml (95% CI: 1315.09 to 1718.07) and only modestly increased in sildenafil-treated diabetic mice: mean change from baseline 227.64 pg/ml (95% CI: 24.40 to 430.88). The LS mean difference between treatments was highly significant, at −1288.94 pg/ml (95% CI: −1575.34 to −1002.54), *P* < 0.001 (Fig. 2E). Neutrophil gelatinase-associated lipocalin (NGAL), strongly expressed in the kidney following ischemic or nephrotoxic injury, also proved to be modulated by sildenafil in DN: STZ + SILD vs. STZ, *P* < 0.05 (Fig. 2F). All this converging evidence supports the assertion that sildenafil protects the kidneys against the progression of diabetic damage.

### Effects of sildenafil on kidney microcirculation assessed by RDU

**Figure 3:**
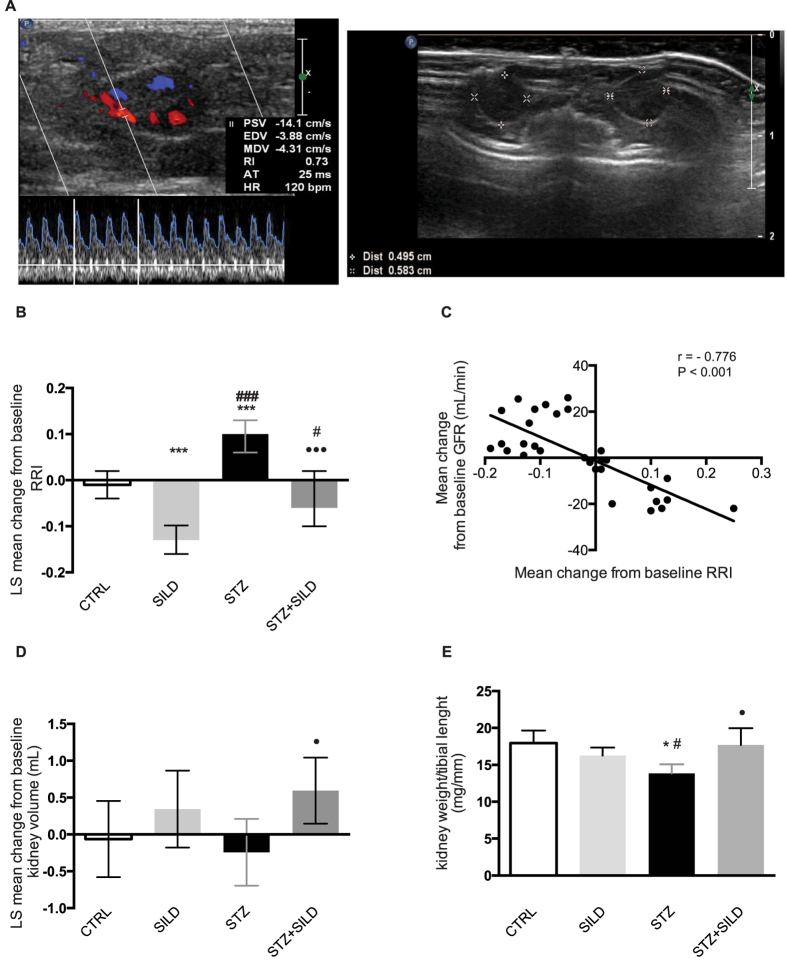
(**A**) representation of RDU image, RRI measurement (on the right) and volume measurement (on the left); (**B**) LS mean change from baseline of RRI in CTRL, SILD, STZ and STZ + SILD mice. Error bars represent upper and lower 95% CI; ***P < 0.001 vs. CTRL; ^#^*P* < 0.05, ^###^*P* < 0.001 vs. SILD; ^•••^*P* < 0.001 vs. STZ. (**C**) correlation between mean change from the baseline in GFR (Y axis) and in RRI (X axis), r = −0.766, *P* < 0.001; (**D**) LS mean change from baseline of renal volume in CTRL, SILD, STZ and STZ + SILD mice. Error bars represent upper and lower 95% CI; ^•^*P* < 0.05 vs. STZ. (**E**) kidney weight/tibial length ratio, mean ± SD, in CTRL, SILD, STZ and STZ + SILD mice. *P < 0.05, vs. CTRL; ^#^*P* < 0.05 vs. SILD; ^•^*P* < 0.05 vs. STZ.

Non-invasive measurement of vascular resistance can be estimated by RRI using RDU (Fig. 3A). RRI of the interlobular arteries was increased in STZ mice compared to CTRL mice and STZ + SILD mice. The mean change in RRI from the baseline was 0.10 (95% CI: 0.06 to 0.13) and −0.06 (95% CI: −0.10 to 0.02) respectively in STZ and STZ + SILD mice, and the treatment-related LS mean change was −0.16 (95% CI: −0.22 to −0.09), *P* < 0.001. No differences were observed between STZ + SILD and CTRL: LS mean change −0.05 (95% CI: −0.11 to 0.01), *P* = 0.120, suggesting that the effect of sildenafil completely reversed the change in RRI. However, a statistically significant difference was observed between STZ + SILD and SILD: LS mean change 0.07 (95% CI: −0.02 to 0.12), *P* = 0.013, (Fig. 3B), suggesting that there is also a vasoactive effect independent of the reversal of diabetic damage. The mean change from the baseline in RRI was inversely correlated with the mean change from baseline in GFR (r = −0.766, *P* < 0.001), (Fig. 3C), even when adjusted for MAP (r = −0.435, *P* = 0.014), suggesting that renal hemodynamics play a leading role in determining renal function response.

*In vivo* evaluation by RDU also showed a reduction in renal volume in STZ treated animals, that was reversed in STZ + SILD: the LS mean difference was 0.83 mL (95% CI: 0.19 to 1.47), *P* = 0.016. There was no difference in renal volume between diabetic mice treated with sildenafil and non-diabetic CTRL or SILD animals (Fig. 3D). The RDU measurement accuracy was confirmed by analysis of renal weight after harvesting. STZ mice showed a significant reduction in the kidney weight/tibial length ratio, whereas STZ + SILD had a ratio comparable with non-diabetic groups, (STZ + SILD: 17.69 ± 2.27 vs. CTRL: 17.94 ± 1.72 g/mm, *P* = 0.874, and vs. SILD: 16.25 ± 1.09 g/mm, *P* = 0.323, and higher than STZ: 13.85 ± 1.23 g/mm, *P* = 0.034), (Fig. 3E).

### Histological changes induced by diabetes correlate with RRI and are prevented by sildenafil

**Figure 4:**
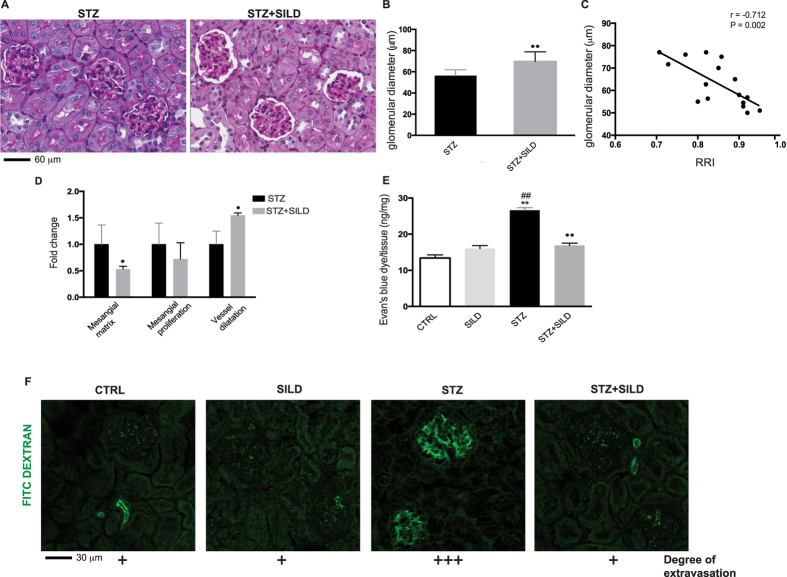
(**A**) representative photomicrographs of PAS-stained kidneys of STZ and STZ + SILD mice,bar represent 60 μm; (**B**) glomerular diameters, mean ± SD, in STZ and STZ + SILD mice. ^••^*P* < 0.01; (**C**) correlation between glomerular diameter (Y axis) and RRI (X axis) at the end of observation period, r = −0.712, *P* = 0.002; (**D**) relative quantification, mean ± SD, of mesangial matrix, mesangial proliferation, vessel dilatation in STZ and STZ + SILD mice, ^•^*P* < 0.05; (**E**) quantification, mean ± SD, of Evan’s Blue dye/mg tissue in CTRL, SILD, STZ and STZ + SILD mice, **P < 0.01 vs. CTRL; ^##^*P* < 0.01 vs. SILD; ^••^*P* < 0.01 vs. STZ. (**F**) rapresentation of vascular permeability express as degree of extravasation (+) by FITC dextran in CTRL, SILD, STZ and STZ + SILD mice, bar represent 30 μm.

Diabetes induction reduced glomerular diameter, produced focal and segmental hyperplasia with diffuse mesangial proliferation and increased mesangial matrix deposition (Fig. 4A–D). At the end of treatment, an inverse correlation was found between RRI and glomerular diameters: r = −0.712, *P* = 0.002 (Fig. 4C). In STZ-only treated mice there were areas of acute tubular degeneration, eosinophilia and proximal tubule basal membrane thickening. Sildenafil treatment did not reduce mesangial proliferation induced by STZ, but reduced mesangial matrix deposition (Fig. 4A,D) (*P* < 0.05). Focal and segmental capillary ectasia was observed in SILD and STZ + SILD groups (Fig. 4A,D). A significant correlation between mean change from baseline of RRI and mesangial matrix deposition score (r = 0.627, *P* = 0.009), was found among groups, suggesting that parenchymal stiffness associated with increased mesangial deposition directly contributes to increased resistance of the interlobular arteries. No change was found between RRI and mesangial proliferation score (r = 0.116, *P* = 0.669). There was an inverse correlation between RRI and glomerular vessel diameter (r = −0.703, *P* = 0.002). Overall, these data indicate that hemodynamic changes induced by diabetes, and reversed by sildenafil, mirror discrete changes in the parenchymal and glomerular structure of the kidney.

### Sildenafil improves microcirculation in the diabetic kidney

Microvascular impairment has an important role in the progression of chronic kidney diseases. Disruption of the endothelium (due to injury or non-renewal) contributes to increased vascular permeability. Vascular leakage was quantified by Evan’s blue dye in kidney tissue. In the kidney of STZ mice, the amount of Evan’s blue dye/mg tissue was higher than in non-diabetic mice (STZ vs. CTRL *P* < 0.01 and vs. SILD *P* < 0.01) and was partially reversed by sildenafil treatment (STZ vs. STZ + SILD *P* < 0.01), (Fig. 4E), suggesting that sildenafil protected against the increased vascular leakage associated with diabetes.

Vascular permeability was also assessed by FITC-dextran. In healthy animals, plasma injection with FITC-dextran labeled the inner lumen of glomerular capillaries. In diabetic animals, a substantial increase in FITC-dextran glomerular extravasation was observed compared to non-diabetic (*P* < 0.05), which was partially reversed by sildenafil (*P* < 0.05), (Fig. 4F).

**Figure 5:**
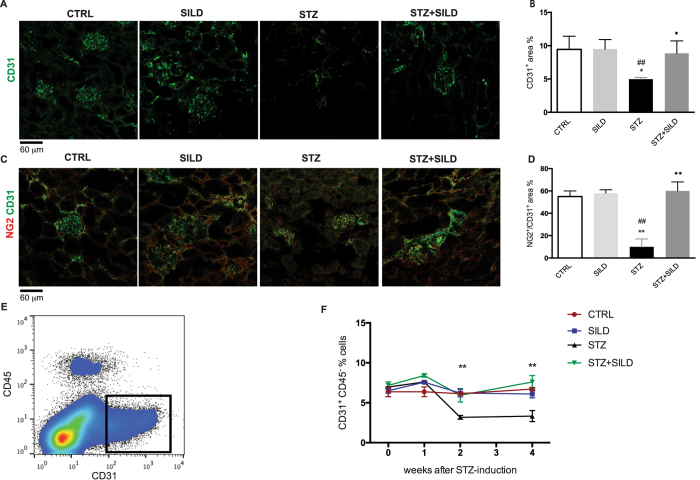
(**A**) Immunofluorescence of CD31^+^ areas in CTRL, SILD, STZ and STZ + SILD mice, bar represent 60 μm; (**B**) Quantification, mean ± SD, of capillary density, expressed as percentage of CD31^+^ area in CTRL, SILD, STZ and STZ + SILD mice. *P < 0.05 vs. CTRL; ^##^*P* < 0.01 vs. SILD; ^•^P < 0.05 vs. STZ; (**C**) Immunofluorescence of CD31^+^ (green) and NG2^+^ (red) areas in CTRL, SILD, STZ and STZ + SILD mice, bar represent 60 μm; (**D**) Quantification, mean ± SD, of pericytes coverage, expressed as percentage of ratio NG2^+^/CD31^+^ area in CTRL, SILD, STZ and STZ + SILD mice. **P < 0.01 vs. CTRL; ^##^*P* < 0.01 vs. SILD; ^••^*P* < 0.01 vs. STZ; (**E**) Representative gating of CD31^+^CD45^−^ cells. (**F**) Percentage of CD31^+^CD45^−^ EC in kidney, before and after STZ induction in CTRL, SILD, STZ and STZ + SILD mice. **P < 0.01 between STZ and CTRL, SILD and STZ + SILD groups.

Since there is a loss of EC in DN^28^, we evaluated the expression of the EC marker CD31. Capillary density as quantified by CD31^+^ area, was reduced in STZ mice compared to non-diabetic mice (STZ vs. CTRL, *P* = 0.019; STZ vs. SILD, *P* = 0.007), but partially restored by sildenafil treatment (STZ vs. STZ + SILD, *P* = 0.024). CD31^+^ area was similar in all non-diabetic and sildenafil treated mice, (Fig. 5A,B). Given the reduction in EC, which might be due to impaired perivascular cell function, we investigated vascular architecture by double staining for CD31 and a pericyte marker, NG2. Pericyte coverage of the endothelium, quantified as percentage of NG2^+^/CD31^+^ area ratio, revealed a disassociation of pericytes in the STZ group that was preserved in STZ + SILD mice (respectively 10 ± 7% vs. 60 ± 8%, *P* < 0.01) to a level comparable to CTRL mice (Fig. 5C,D). To better characterize the pattern of CD31^+^ loss, we analyzed CD31^+^ cells in renal tissue by flow cytometry at different time points (Fig. 5E). This revealed a reduction of CD31^+^ EC in STZ mice, which was already detectable from the second week after diabetes induction (week 2: STZ vs. CTRL, *P* = 0.002; STZ vs. SILD, *P* = 0.004; STZ vs. STZ + SILD, *P* = 0.005) and increased thereafter, suggestive of EC death. Sildenafil prevents EC loss along the entire observation time (week 4: STZ vs. CTRL, *P* = 0.005; STZ vs. SILD, *P* = 0.008; STZ vs. STZ + SILD, *P* = 0.007), (Fig. 5F). Collectively, these data indicate that sildenafil protected the renal microcirculation by favoring vascular integrity in diabetic mice.

### Sildenafil upregulates BMP7 and downregulates miR-22 in diabetic kidney

**Figure 6:**
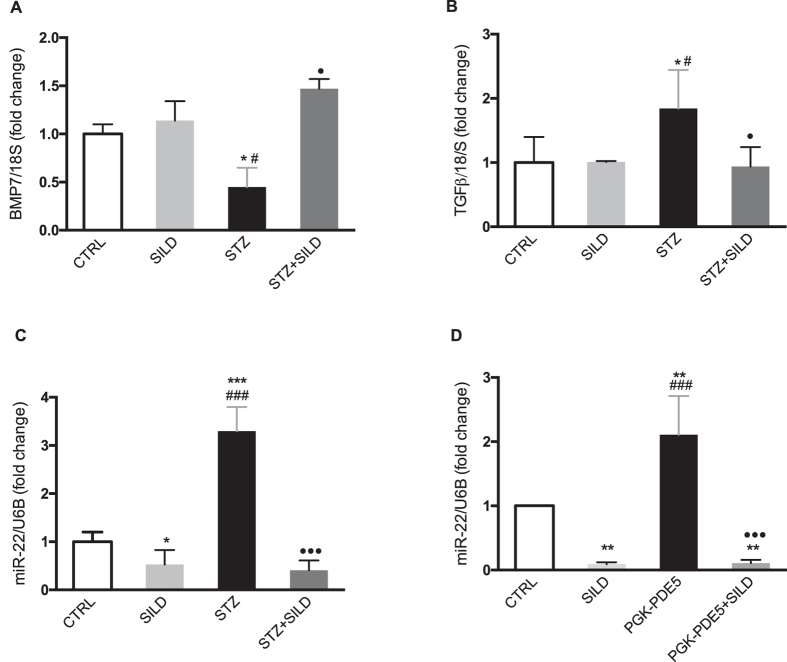
(**A**) Relative quantification, mean ± SD, of BMP7/18S by qPCR in kidney of CTRL, SILD, STZ and STZ + SILD mice. *P < 0.05 vs. CTRL, ^#^*P* < 0.05 vs. SILD, ^•^*P* < 0.05 vs. STZ; (**B**) Relative quantification, mean ± SD, of TGFβ/18S by qPCR in kidney of CTRL, SILD, STZ and STZ + SILD mice. *P < 0.05 vs. CTRL, ^#^*P* < 0.05 vs. SILD, ^•^*P* < 0.05 vs. STZ; (**C**) Relative quantification, mean ± SD, of miR-22/U6B by qPCR in kidney of CTRL, SILD, STZ and STZ + SILD mice. *P < 0.05 and ***P < 0.001 vs. CTRL, ^###^*P* < 0.001 vs. SILD, ^•••^*P* < 0.001 vs. STZ; (**D**) Relative quantification, mean ± SD, of miR-22/U6B by qPCR in HUVEC untreated, treated with sildenafil, PKG-PDE5-transduced, PGK-PDE5-transduced and treated with sildenafil. **P < 0.01 vs. CTRL, ^###^*P* < 0.001 vs. SILD, ^•••^*P* < 0.001 vs. PGK-PDE5.

Bone morphogenetic protein-7 (BMP7) is known to be a critical molecule in inducing angiogenesis, protecting EC from apoptosis and antagonizing TGF-β-dependent fibrosis^29^. We observed a BMP7 downregulation in diabetic kidney (*P* < 0.05) and, conversely, an increased expression of BMP7 after sildenafil treatment (*P* < 0.05), (Fig. 6A). This was also associated with downregulation of BMP7-antagonist TGF-β (*P* < 0.05), (Fig. 6B). It was previously demonstrated that BMP7 is regulated by miR-22^30^. In light of the observed downregulation of miR-22 in adipose tissue after sildenafil treatment^31^, we decided to verify whether miR-22 underwent a similar pattern of downregulation in kidney. To this end, we examined miR-22 expression by qPCR, finding that it was downregulated by sildenafil ~3 fold (*P* < 0.001), (Fig. 6C). This supports the nephroprotective role of PDE5i through the miR-22/BMP7 axis. We also extended our *in vivo* observations, investigating whether the PDE5 pathway was directly involved in miR-22 expression in endothelial cells (HUVEC). We engineered HUVEC to trigger PDE5 hyper-expression and we found that miR-22 was highly upregulated (*P* < 0.01), with downregulation after sildenafil treatment occurring in both transduced (*P* < 0.001) and non-transduced cells (*P* < 0.01), (Fig. 6D). This indicates both a strong association between PDE5 and miR-22 expression and the ability of sildenafil to induce a marked reduction in this damage marker.

## Discussion

Experimental and clinical data support the hypothesis that PDE5is could act as nephroprotective agents and current interest has shifted from their cardioprotective^32^,^33^,^34^ to their nephroprotective^13^,^14^,^15^,^16^,^17^,^18^,^35^,^36^ properties. In a recent clinical trial, a novel long-acting PDE5i, PF-00489791, was effective in reducing albuminuria in diabetic patients stably treated with angiotensin-converting enzyme inhibitors (ACEi) or angiotensin receptor blockers (ARBs). This reduction occurred without any changes in GFR and independently of changes in blood pressure^37^. This prompted us to investigate the mechanisms involved in sildenafil’s protection in DN. We used a high-dose STZ model after pretreatment with a sodium/glucose cotransporter inhibitor, phlorizin, which reduces the risk of any acute nephrotoxic effects of STZ, while maintaining the known β-cell toxicity^26^. We found that sildenafil prevented renal function from worsening, as shown by GFR and ACR, improved microcirculation, by means of *in vivo* assessment of RRI, and slowed the progression of DN, specifically targeting mesangial matrix expansion, capillary and glomerular diameter. We then provided molecular insights into involvement of the miR-22 and its direct target BMP7 in modulating angiogenesis and vascular stability in the diabetic kidney.

Previous studies clearly demonstrated that a rise in cGMP levels and enhanced eNOS expression following PDE5 inhibition is essential for the maintenance of renal perfusion and glomerular filtration. However, the direct effects of NO in glomerular hemodynamics include preglomerular vasodilatation^38^, therefore PDE5 inhibition would be expected to aggravate, rather than improve, the glomerular hypertension and hyper-filtration triggering progressive glomerulosclerosis in DN. In addition, blood pressure lowering agents such as ACEi or ARBs have been associated with short-term lowering of GFR^39^, in contrast with other studies^37^, and in this study we demonstrate that GFR and ACR are modulated by sildenafil independently of MAP changes. This seems to suggest that the nephroprotective effects of PDE5i are not secondary to a reduction in systemic hypertension, but rather are exerted through a modulation of intraglomerular hemodynamics.

In line with its protective effects we also found a reduced expression of nephropathy biomarkers such as NGAL and suPAR in sildenafil treated mice. A rise in suPAR increases glomerular permeability, leading to a cascade of events that result in focal segmental glomerulosclerosis; suPAR is thus emerging as a new biomarker for chronic kidney disease^40^.

An advantage of this study is its *in vivo* assessment of interlobular renal arteries by RDU, which is gaining momentum in the early identification of morphologic and hemodynamic renal changes in diabetic patients^41^. Increased RRI is correlated with renal dysfunction^42^, T2DM and chronic nephropathies^43^,^44^, and, in the absence of macrovascular disease, reflects the integrity of the renal microcirculation^45^. PDE5is have been shown to offer vascular protection in hypertension^46^, however to the best of our knowledge no studies have used RDU to investigate *in vivo* the effect of PDE5is in a mouse model of DN. As seen in diabetic patients^24^, RRI inversely correlated with GFR. Increased RRI can reflect changes in intraglomerular hemodynamics, but also a deterioration in tissue architecture due to a reduction of intravessel area and tissue stiffness and consequent increase vascular resistance^42^. We clearly observed an increase of RRI in diabetic mice; this was correlated with histopathological changes and was reduced by sildenafil. These observations point to the direct modulation of renal microcirculation by PDE5i.

Sildenafil treatment determined an improvement in the renal vascular rarefaction induced by diabetes and also limited EC-pericytes detachment. It has been postulated that PDE5i ameliorated endothelial function^19^,^20^. It was recently demonstrated that sildenafil recovered the endothelial surface and eliminated oxidative stress in an experimental model of hypertension, reducing blood pressure and improving the endothelium-dependent relaxation^35^,^47^.

BMP7 plays a pivotal role in kidney homeostasis by antagonizing the TGFβ-induced profibrotic signals and counteracting the TGFβ-induced pericytes-to-myofibroblast transition^29^. Its anti-fibrotic action could restore vascular stability and limit capillary rarefaction. The beneficial effects of BMP7 signaling in diabetic nephropathy were confirmed in pharmacological transgenic mice models overexpressing BMP7 in podocytes and proximal tubules^48^. Recombinant BMP7 therapy has been shown to inhibit fibrosis progression in a variety of mouse models of renal fibrosis, including the STZ^49^ diabetic mice. In this study, we demonstrate that sildenafil increases BMP7 expression in diabetes and that such effect occurs alongside the downregulation of miR-22, a known regulator of BMP7. The enhanced BMP7 expression seen with sildenafil administration could explain the vessel structure preservation and limited mesangial expansion occurring in diabetic mice treated with PDE5i. MiR-22 is known to directly target BMP7, and deletion of miR-22 significantly attenuated renal fibrosis^30^. MiR-22 is also involved in blood pressure regulation. *In vivo* administration of antagomir of miR-22 to spontaneously hypertensive rat reduced blood pressure and revealed a new avenue for treatment of hypertension^50^. We recently demonstrated that sildenafil determined a downregulation of circulating miR-22 in diabetic patients and in adipose tissue of db/db mice^31^. In the present study, we confirmed the downregulation of miR-22 by sildenafil in diabetic kidney and upregulation of its target BMP7, associated with tissue protection. Importantly, we provide a confirmation of involvement of PDE5 signaling on miR-22 expression.

However, this study has some limitations. First, we cannot demonstrate how sildenafil modulates the elements regulating miR-22 gene expression. Second, the relatively short observation period could not discern whether sildenafil acts on DN development or progression; long-term studies are warranted to address this issue. Finally, further studies on DN under controlled genetic and environmental conditions mouse models and/or in combination with hypoglycemic therapies could help to better investigate the therapeutic benefit of PDE5is.

This study extends our previous findings on the effect of PDE5is on diabetic vascular complication, supporting their therapeutic role in slowing the natural progression of kidney damage during DN.

Our results demonstrate that metabolic, hemodynamic and epigenetic factors combine to preserve vessel structure and function in DN and uncover the novel role of PDE5i/miR-22 regulatory relationship, which helps to preserve BMP7 homeostasis in the kidney. Translational confirmatory findings will reveal novel treatment strategies for diabetic nephrovascular disease that could be immediately transferred to clinical practice.

## Additional Information

**How to cite this article**: Pofi, R. *et al*. Phosphodiesterase-5 inhibition preserves renal hemodynamics and function in mice with diabetic kidney disease by modulating miR-22 and BMP7. *Sci. Rep.* **7**, 44584; doi: 10.1038/srep44584 (2017).

**Publisher's note:** Springer Nature remains neutral with regard to jurisdictional claims in published maps and institutional affiliations.
